# Chemistry and Selective Tumor Cell Growth Inhibitory Activity of Polyketides from the South China Sea Sponge *Plakortis* sp.

**DOI:** 10.3390/md15050129

**Published:** 2017-05-03

**Authors:** Jiao Li, Cui Li, Raffaele Riccio, Gianluigi Lauro, Giuseppe Bifulco, Tie-Jun Li, Hua Tang, Chun-Lin Zhuang, Hao Ma, Peng Sun, Wen Zhang

**Affiliations:** 1 Research Center for Marine Drugs, School of Pharmacy, Second Military Medical University, 325 Guo-He Road, Shanghai 200433, China; lijiao_2012@126.com (J.L.); licuiwan@163.com (C.L.); ltj204@163.com (T.-J.L.); tanghua0309@126.com (H.T.); zclnathan@163.com (C.-L.Z.); Tobi_hao@outlook.com (H.M.); sunpeng78@126.com (P.S.); 2 Dipartimento di Farmacia, Universita’ di Salerno, Via Giovanni Paolo II 132, 84084 Fisciano (SA), Italy; riccio@unisa.it (R.R.); glauro@unisa.it (G.L.); bifulco@unisa.it (G.B.); 3 Science and Research Laboratory, Longhua Hosptial, Shanghai University of Traditional Chinese Medicine, 725 South Wanping Road, Shanghai 200032, China

**Keywords:** polyketide, sponge, *Plakortis* sp., quantum mechanical calculation, selective activity

## Abstract

Simplextone E (**1**), a new metabolite of polyketide origin, was isolated with eight known analogues (**2**–**9**) from the South China Sea sponge *Plakortis* sp. The relative configuration of the new compound was elucidated by a detailed analysis of the spectroscopic data and quantum mechanical calculation of NMR chemical shifts, aided by the newly reported DP4+ approach. Its absolute configuration was determined by the TDDFT/ECD calculation. Simplextone E (**1**) is proven to be one of the isomers of simplextone D. The absolute configuration at C-8 in alkyl chain of plakortone Q (**2**) was also assigned based on the NMR calculation. In the preliminary in vitro bioassay, compounds **6** and **7** showed a selective growth inhibitory activity against HCT-116 human colon cancer cells with IC_50_ values of 8.3 ± 2.4 and 8.4 ± 2.3 μM, corresponding to that of the positive control, adriamycin (IC_50_ 4.1 μM). The two compounds also showed selective activities towards MCF-7 human breast cancer and K562 human erythroleukemia cells while compound **3** only displayed weak activity against K562 cells.

## 1. Introduction

Sponges of the genus *Plakortis* are known to produce structurally diverse and pharmacologically active polyketides [1]. This family of polyketides are commonly characterized with a cyclic peroxide [2,3,4,5,6], a penta-lactone [7,8,9,10,11,12,13], or a furano ring [14,15,16] as core structure, and a flexible alkyl chain. A small number of polyketides have a simple linear structure [17,18]. Cyclic peroxides with 1,2-dioxane and 1,2-dioxolane ring systems are the most abundant metabolites, showing remarkable biological activities such as cytotoxic [3,5], antiparasitic [14], antibacterial [2,19] and antimalarial [20] effects. Polyketides with lactone fragment, i.e., plakortones A-G, are the second most prominent metabolites in these sponge-derived polyketides, exhibiting cardiac SR-Ca^2+^-pumping ATPase activating and in vitro cytotoxic activities [7,8,9]. The intrigoing structures and broad spectrum of bioactivities of the metabolites attracted attention on the chemical synthesis of such a cluster of molecules [21,22,23,24,25,26,27,28,29]. Also, the high flexibility of aliphatic chains or rings of these polyketides leads to a challenge in the absolute configuration identification. The absolute configurations were determined at an early stage mainly by chemical methods, such as chemical degradation [30] and total synthesis [29,31,32]. In recent years, the calculating of NMR parameters provided a strong and convenient approach for the assignment of relative or absolute configuration of these highly flexible systems [33,34,35].

**Figure 1:**
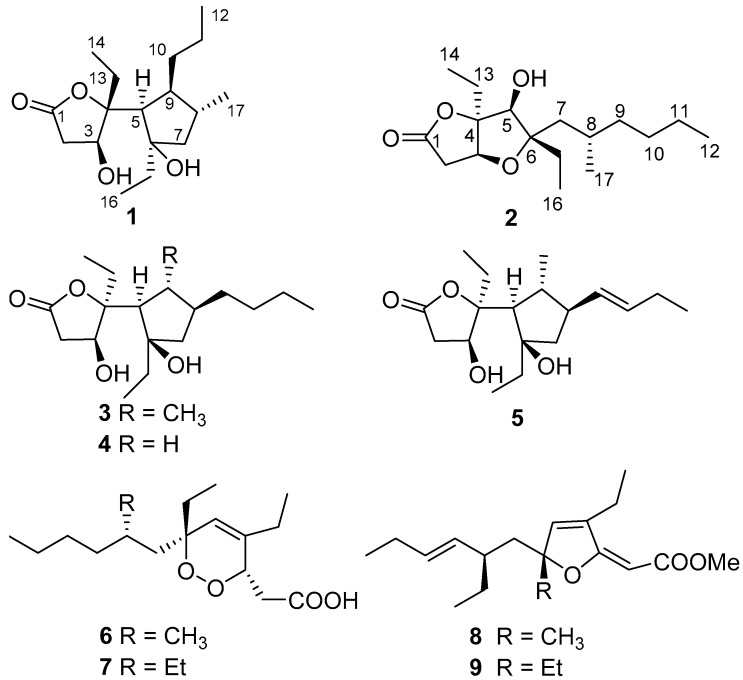
Structures for compounds **1**–**9**.

In the course of our continuing search for novel and bioactive secondary metabolites from marine invertebrates [36,37], we reinvestigated the marine sponge *Plakortis* sp., collected from the South China Sea, leading to the isolation of a new polyketide, simplextone E (**1**), together with eight known analogues (**2**–**9**) (Figure 1), namely lactone derivatives plakortone Q (**2**) [13], simplextones A-C (**3**–**5**) [11,12], peroxide derivatives methyl (3*S*,6*R*,8*S*)-4,6-diethyl-3,6-epidioxy-8-methyldodeca-4-enoate (**6**) [16], haterumadioxin B (**7**) [38], furanylidene derivatives spongosoritin A (**8**) [15,39], and 6-desmethyl-6-ethylspongosoritin A (**9**) [40,41]. The structures of these compounds were elucidated by extensive spectroscopic analysis and compared with the reported data. To assign the absolute configuration of compounds **1** and **2**, we applied quantum mechanical calculation of NMR chemical shifts and ECD. Here, we report the isolation, structural determination, and tumor cell growth inhibitory activity bioactivity of these compounds.

## 2. Results and Discussion

### 2.1. Isolation and Stereostructural Determination

Freshly collected specimens of *Plakortis* sp. were immediately frozen at −20 °C and stored at this temperature before extraction. Frozen material was cut into small pieces and extracted with acetone. The diethyl ether soluble portion of the acetone extract was subjected to repeated column chromatography on silica gel, Sephadex LH-20, and RP-HPLC to afford nine polyketides **1**–**9**. By extensive spectroscopic analysis combined with careful comparison with the reported data, the structures of the known compounds were determined as lactone derivatives plakortone Q (**2**) [13], simplextones A-C (**3**–**5**) [11,12], peroxide derivatives methyl (3*S*,6*R*,8*S*)-4,6-diethyl-3,6-epidioxy-8-methyldodeca-4-enoate (**6**) [16] and haterumadioxin B (**7**) [38], furanylidene derivatives **8** [15] and **9** [40,41]. Plakortone Q (**2**) and simplextones A-C (**3**–**5**) were once reported from the South China Sea sponge *P. simplex*, while peroxide derivatives **6** and **7** were previously obtained from *P. angulospiculatus* and *P. lita*, respectively. Furanylidene derivatives **8** and **9** were previously isolated from *P. angulospiculatus* and *P. halichondrioides*, respectively. Compounds **3**–**5** were reported to have moderate cytotoxicity [11,12], compound **6** showed potent antileishmanial activity [16], compound **8** exhibited significantly activity in brine shrimp assay [15], and compound **9** exhibited potent cytotoxic activities against the HeLa and K562 cell lines [12]. 

Simplextone E (**1**) was isolated as a colorless oil. The molecular formula of **1** was determined as C_17_H_30_O_4_ from the HRESIMS data (316.2485 [M + NH_4_]^+^) and it required three degrees of unsaturation. The IR spectrum of **1** indicated the presence of hydroxy (3438 cm^−1^) and ester carbonyl (1749 cm^−1^) groups. The ^13^C NMR and DEPT data (Table 1) displayed seventeen carbon signals, including one *sp*^2^ carbon atom (one ester carbonyl group) at a lower field and sixteen *sp*^3^ carbon atoms at a higher field (four methyl, six methylenes, three methines, one oxygenated methine, two oxygenated tertiary carbons), accounting for one double bond equivalents. The remaining double bond equivalents were due to the presence of two rings in the molecule.

**Figure 2:**
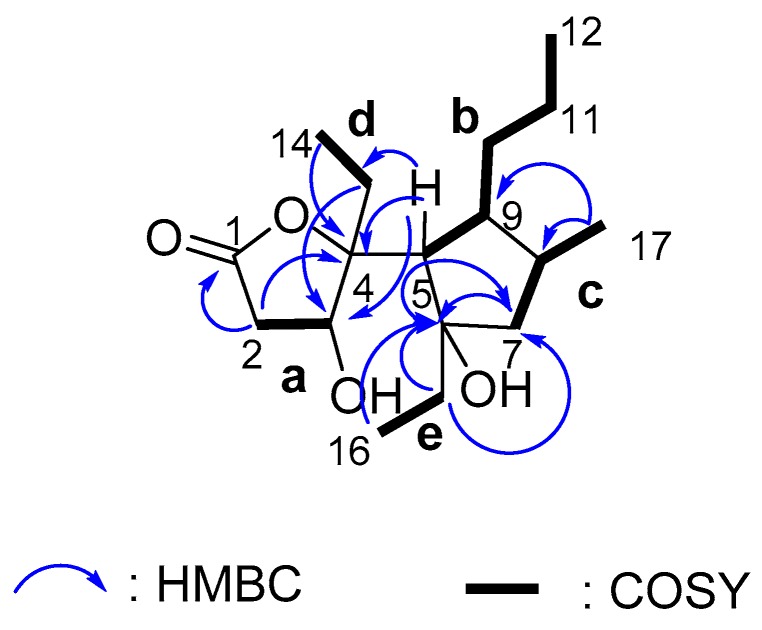
Key HMBC (arrow H→C) and ^1^H-^1^H COSY (bond) correlations for compound **1**.

By interpreting the ^1^H-^1^H COSY correlations, it was possible to establish five partial structures of consecutive proton systems as **a**-**e** (H-2/H-3, H-5/H-9/H-10/H-11/H-12, H-7/H-8/H-17, H-13/H-14, and H-15/H-16, Figure 2). The HMBC correlations from Me-17 to C-7, C-8 and C-9 revealed the linkage of moieties **b** and **c** by C-8-C-9 bond. The HMBC correlations from H-5, H-7, and Me-16 to C-6 and from H-5 and H-15 to C-7 give a connection of fragments **b**, **c**, **e** by the oxygenated tertiary carbon C-6, leading to the formation of a cyclopentane ring. The HMBC correlations from H-2 and Me-14 to C-4, and from H-5 to C-3, C-4, and C-13 led the connection of **a**, **d**, and the cyclopentane ring by another oxygenated tertiary carbon C-4. The HMBC correlation from H-2 to C-1 indicated the ester carbonyl group was connected to fragment **a**. The remaining one double bond equivalent in the molecule allowed for the linkage from the ester carbon C-1 to the tertiary carbon C-4 by an oxygen bridge to form a γ-butyrolactone ring. The planar structure of simplextone E (**1**) was then established. The assigned planar structure of **1** was identical to that of simplextone D [12], which was previously obtained from the Chinese sponge *P. simplex*. However, a big difference was observed regarding the chemical shift values for H_3_-14, C-8 and C-9, indicating a different stereochemistry.

**Figure 3:**
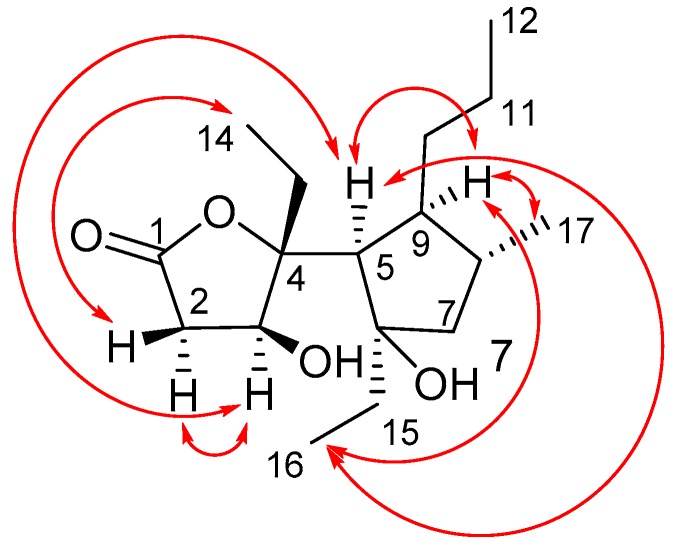
Key NOESY correlations for compound **1**.

The relative configuration of **1** was determined by 1D and 2D NOE experiments (Figure 3). The distinct NOESY cross peak between H-3 and H-5 indicated the same orientation of these protons. This observation was in agreement with the NOE between H-3 and H-2α and H-5 in the NOE difference spectrum (Figure S9). The NOE correlation Between H-2β and Me-14 in the NOESY spectrum supported the above conclusion. On the other hand, the NOE correlations of Me-16 with H-5 and H-9, and of H-9 with Me-17 indicated that these groups were oriented on the same side of the cyclopentane ring (Figure 3). Thus, the relative configurations of γ-butyrolactone and cyclopentane ring were determined, respectively.

**Figure 4:**
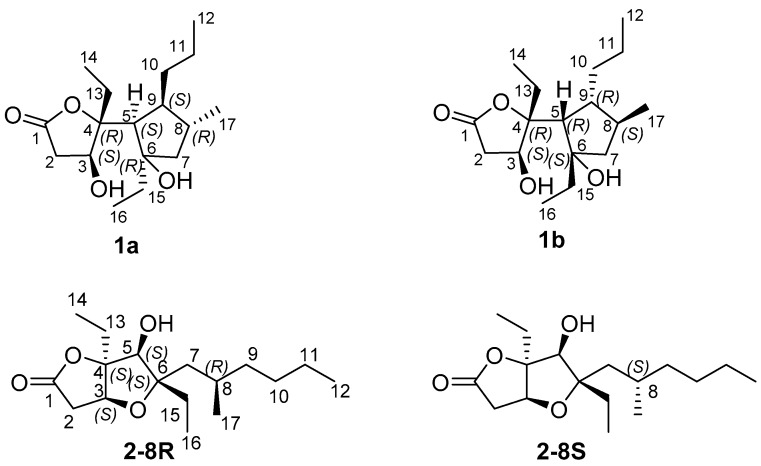
Possible relative stereostructures for **1** and **2**.

**Figure 5:**
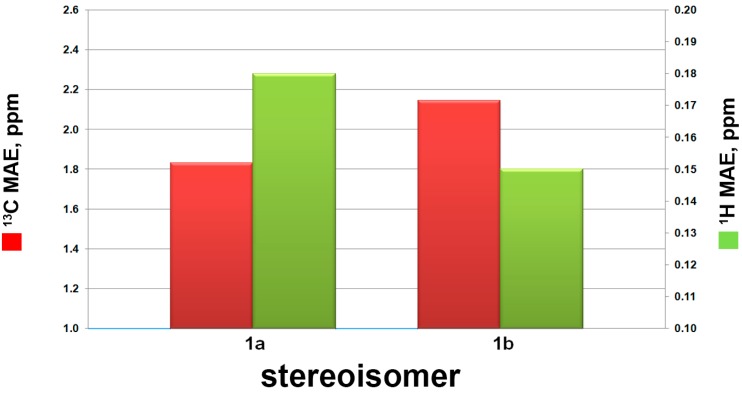
^13^C (red bars) and ^1^H (green bars) mean absolute errors (MAE) histograms related to compounds **1a-b**, as indicated in Table 2.

However, the relative configuration of the two conjoined bicyclic systems could not be identified unambiguously by NMR data because of the free rotating nature of C-4/C-5 single bond. Hence, quantum mechanical calculation of ^13^C NMR chemical shifts and DP4+ method were applied to determine the relative configuration of **1**. There were two possible candidate configurations of **1** (**1a** and **1b** in Figure 4). An extensive conformational search at the empirical level was carried out for **1a**-**1b** (see Computational Details, Experimental Section). Then, the obtained conformers were submitted to a geometry and energy optimization phase at the density functional level (DFT), and afterwards ^13^C and ^1^H NMR chemical shifts were predicted for **1a**-**1b** at the DFT. Mean absolute error (MAE) values were used to compare calculated and experimental ^13^C and ^1^H NMR chemical shifts (see Table 2 and Figure 5). Although mean absolute errors (MAE) values of ^1^H NMR and ^13^C NMR were not in accordance, ^13^C NMR MAE values clearly pointed out **1a** as the most probable isomer (MAE values: 1.83 for **1a**, 2.14 for **1b**) (Figure 5). To unambiguously assign the relative configuration of **1**, we then computed the DP4+ probabilities [42] (Table 2) for the two possible considered stereoisomers (**1a**-**1b**). Specifically, the best DP4+ performance in predicting the correct stereochemistry of organic compounds has been associated to the combination of both ^1^H and ^13^C chemical shift data. In fact, when ^1^H/^13^C DP4+ probabilities point to two different stereoisomers causing the uncertainty in assigning the relative configuration, the combination of both has been shown to determine a clear enhancement of DP4+ performance, indicating that data from the two nuclei should be accounted for, when available. In our case, all data DP4+ corroborated **1a** as the most probable relative stereoisomer, and accordingly, the relative configuration of **1** was established as 3S*, 4R*, 5S*, 6R*, 8R*, 9S*.

**Figure 6:**
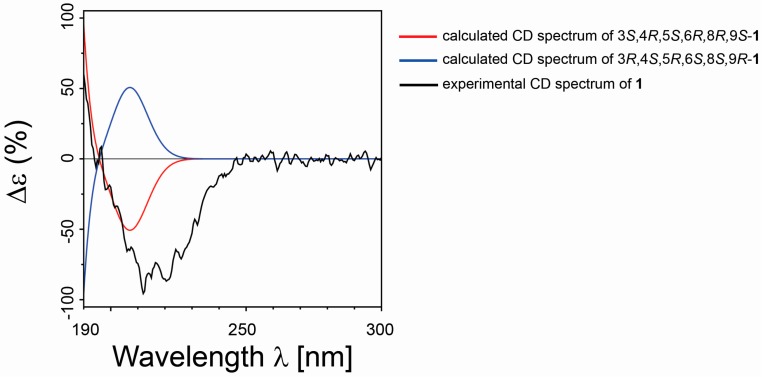
Comparison between the calculated ECD spectra (for 3*S*, 4*R*, 5*S*, 6*R*, 8*R*, 9*S*/3*R*, 4*S*, 5*R*, 6*S*, 8*S*, 9*R*, in red and blue, respectively) and experimental ECD spectrum (in black) of **1**.

Once the relative configuration of **1** was proposed, we then determined the absolute configuration applying to the TDDFT/ECD calculation [37,43]. The conformers of **1a** produced in the above reported step of NMR QM calculations were then submitted to a further optimization step at the DFT, reproducing experimental solvent effects (CH_3_CN) and using the integral equation formalism version of the polarizable continuum model (IEFPCM). We built the final ECD spectrum for the identified diastereoisomer, considering the influence of each conformer on the total Boltzmann distribution and taking into account the relative energies. The experimental ECD curve aligned well with the calculated ECD curve for 3*S*, 4*R*, 5*S*, 6*R*, 8*R*, 9*S* isomer (Figure 6), and this allowed us to assign the absolute configuration for compound **1**.

**Figure 7:**
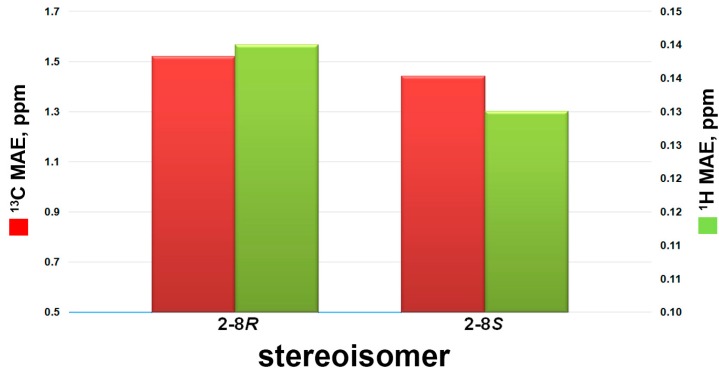
^13^C (red bars) and ^1^H (green bars) mean absolute errors (MAE) histograms related to compounds **2-8*R*** and **2-8*S***, as indicated in Table 3.

**Figure 8:**
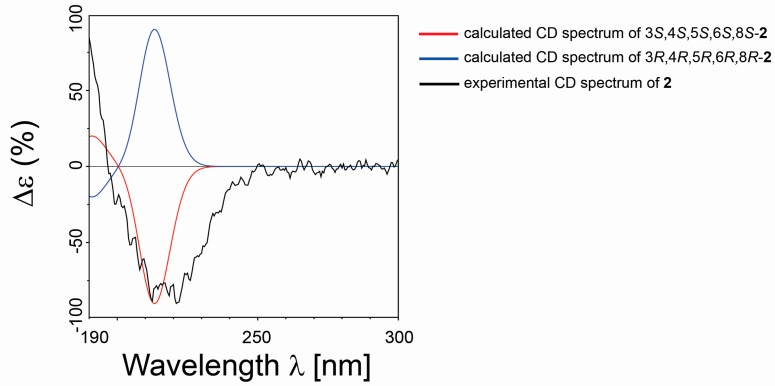
Comparison between the calculated ECD spectrum (3*S*, 4*S*, 5*S*, 6*S*, 8*S*/3*R*, 4*R*, 5*R*, 6*R*, 8*R* in red and blue, respectively) and experimental ECD spectrum (in black) of **2**.

Compound **2** was isolated as a colorless oil. The HRESIMS and NMR spectroscopic data of **2** reveal the identical structure of plakortone Q, recently obtained from the Chinese sponge *Plakortis simplex* [13]. However, the absolute configuration of C-8 in the alkyl chain was not determined in the literature. We then applied the above reported method to assign the absolute configuration of C-8. There were two possible absolute configurations of C-8, 8*R* and 8*S*. Thus, there were two candidate stereostructures of **2** (**2-8*R*** and **2-8*S*** in Figure 4). Therefore, the configuration of C-8 of **2** was assigned as *S* by using the quantum mechanical calculation of ^1^H and ^13^C NMR chemical shifts (^13^C MAE values: 1.52 for **2-8*R***, 1.44 for **2-8*S***; ^1^H MAE values: 0.14 for **2-8*R***, 0.13 for **2-8*S***, Figure 7), this was also confirmed by the analysis of all data DP4+ probabilities as previously mentioned in **1** (Table 3). Finally, the TDDFT/ECD calculations were also performed starting from conformers of **2-8*S*** produced in the QM step of computation of the NMR chemical shift data, and they were submitted to another round of geometry optimization at the DFT in CH_3_CN IEFPCM. The obtained curves for the two possible enantiomeric species superimposed with the experimental one (Figure 8) disclosed 3*S*, 4*S*, 5*S*, 6*S*, 8*S* as the absolute configuration of **2**, thus conforming the stereochemistry pattern at the bicyclic portion as previously reported [13].

### 2.2. In Vitro Evaluation of Cytotoxic Activity

**Figure 9:**
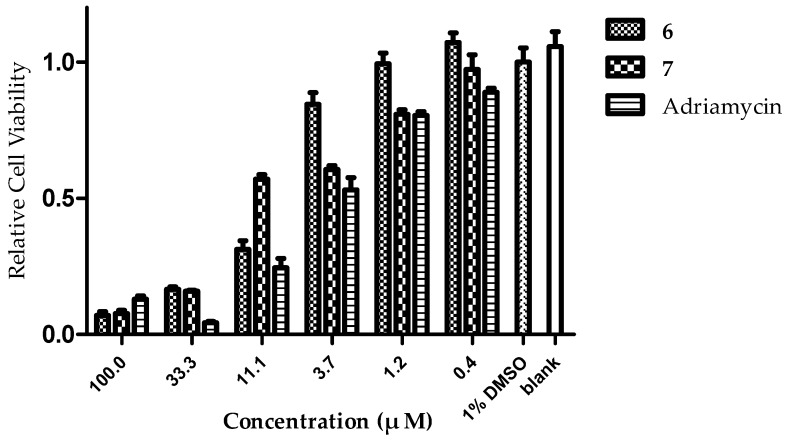
Histograms of relative cell viability percentage for compounds **6** and **7** against HCT-116 cell lines.

Compounds **1**–**9** were evaluated in vitro for their tumor cell growth inhibitory activity against HCT-116, SMMC-7721 and MCF-7 cell lines (Table 4, Figure 9, Figure S10 and S11). Compounds **6** and **7** showed potent growth inhibitory effect toward HCT-116 cells with IC_50_ values of 7.2 ± 1.1 and 7.4 ± 1.7 μM, respectively. Compound **6** also showed activity towards K562 and MCF-7 cells whereas compound **7** only showed activity towards K562. In addition, compound **3** displayed weak activity against MCF-7 cells (Table 4). All these compounds showed no growth inhibitory activity against SMMC-7721 cell lines (IC_50_ values > 50 μM). The selective activity of **6** and **7** towards HCT-116 colon cancer cells continued.

## 3. Materials and Methods

### 3.1. General Experimental Procedures

Column chromatography (CC) was performed on silica gel (200–300; 400–500 mesh, Yantai, China), RP silica gel (43–60 μm, Merck, Darmstadt, Germany) and Sephadex LH-20 (GE Healthcare Bio-Sciences AB, SE-751 84 Uppsala, Sweden). TLC was performed on precoated silica gel plates (HSGF-254, Yantai, China) and RP silica gel (RP-18 F254, Macherey-Nagel, Düren, Germany). Anisaldehyde-sulphuric acid reagent was used for detecting spots on TLC. An Agilent 1100 system (refractive index detector, YMC Pack ODS-A column (10 × 250 mm, 5 μm)) was put to use to carry on HPLC purification. The NMR data were measured on Bruker DRX 400 at 300 K. Parts per million (δ) was used to report chemical shifts applying residual CDCl_3_ signal as an internal standard (^1^H NMR: δ_H_ 7.26 ppm, ^13^C NMR: δ_C_ 77.00 ppm, Hz for coupling constants (*J*)). Autopol IV polarimeter was used to record the optical rotations in CH_2_Cl_2_ at the sodium D line (590 nm). Nexus 470 FT-IR spectrophotometer (Nicolet, Ramsey, MN, USA) was applied to measure the infrared spectra of compounds in thin polymer films and peaks are reported in cm^−1^. Varian Cary 100 UV-Vis spectrophotometer was adopted to record UV absorption of compounds and wavelengths are reported in nm. JASCO J-810 circular dichroism spectropolarimeter was used to measure circular dichroism spectra. A Q-TOF micro mass spectrometer was utilized to measure the HRESIMS, and the reference compound was sodium iodide dissolved in isopropyl alcohol (2 mg/mL).

### 3.2. Animal Material

The marine sponge *Plakortis* sp. (1.1 kg, dry weight) was collected near Yongxing Island in the South China Sea in November 2011 and identified by Yalan Zhou. A voucher specimen (LG-10) was deposited in the Second Military Medical University, Shanghai, China.

### 3.3. Extraction and Isolation

The frozen specimen (1.1 kg, dry weight) was cut into small pieces and extracted ultrasonically with acetone (3 L × 3) and MeOH (3 L × 2), respectively. The organic extracts were concentrated under a vacuum to give a residue, which was partitioned between H_2_O and diethyl ether to afford 13.8 g of an Et_2_O extract. The Et_2_O extract was subjected to column chromatography on silica gel to give seven fractions, using petroleum ether and EtOAc (from 100:1 to 0:100) as eluent. Fraction 1 was chromatographed on a silica gel column eluting with a petroleum ether/acetone solvent gradient system (from 100:1 to 0:1), to give 10 subfractions (Fr.1-1 to Fr.1-10). Fr.1-8 (1.08 g) was further subjected to a Sephadex LH-20 chromatography column eluting with CH_2_Cl_2_/MeOH (1:1), to afford four subfractions (Fr.1-8-1 to Fr.1-8-4). Fr.1-8-3 (639.5 mg) was subjected to repeated column chromatography on normal-phase (gradient petroleum ether/acetone, 15:1) and reversed-phase silica gel (gradient MeOH/H_2_O, from 3:7 to 10:0), followed by semi-preparative HPLC (YMC Pack ODS-A, 5 μm, 250 × 10 mm) to yield **1** (1.6 mg, MeOH/H_2_O 70:30, 1.5 mL/min, *t*_R_ = 34.0 min), **3** (15.5 mg, CH_3_CN/H_2_O, 60:40, 1.5 mL/min, *t*_R_ = 67.4 min), **4** (6.1 mg, CH_3_CN/H_2_O, 60:40, 1.5 mL/min, *t*_R_ = 48.8 min), **5** (6.4 mg, CH_3_CN/H_2_O, 60:40, 1.5 mL/min, *t*_R_ = 50.9 min), **6** (5.4 mg, CH_3_CN/H_2_O, 90:10, 1.5 mL/min, *t*_R_ = 24.5 min), **7** (0.8 mg, MeOH/H_2_O, 85:15, 1.5 mL/min, *t*_R_ = 42.6 min), **8** (1.1 mg, MeOH/H_2_O, 80:20, 2 mL/min, *t*_R_ = 33.3 min), **9** (15.7 mg, MeOH/H_2_O, 80:20, 1.5 mL/min, *t*_R_ = 55.5 min). Fraction 2 was separated by Sephadex LH-20 chromatography column (CH_2_Cl_2_/MeOH, 2:1) and normal-phase (gradient petroleum ether/acetone, from 15:1 to 3:2), followed by semi-preparative RP-HPLC (YMC Pack ODS-A, 5 μm, 250 × 10 mm), to yield **2** (5.0 mg, MeOH/H_2_O 70:30, 1.5mL/min, *t*_R_ = 63.0 min).

#### Simplextone E (**1**):

Colorless oil; ${\left[\alpha \right]}_{25}^{5}$ = **−**7.8 (*c* 0.13, CH_2_Cl_2_); UV (CH_3_CN) λmax (log ε) 197 (3.07) nm; CD (CH_3_CN, *c* 1.7 × 10^−4^) λ_max_ (Δε) positive below 190 nm, 216 (−0.86) nm; IR (film) ν_max_ 3438, 2958, 2928, 2864, 1749, 1461, 1259, 1061, 963, 801 cm^−1^; ^1^H NMR and ^13^C NMR spectroscopic data, see Table 1; HRESIMS *m*/*z* 316.2485 [M + NH_4_]^+^, calcd. for C_17_H_34_NO_4_, 316.2482. 

### 3.4. Computational Details

The chemical structures of compounds **1a**, **1b**, **2-8*R***, **2-8*S*** were built by Maestro 10.2 [44], and optimized by MacroModel 10.2 [45] with the OPLS force field [46] and the Polak-Ribier conjugate gradient algorithm (PRCG, maximum derivative less than 0.001 kcal/mol). Conformational search rounds for the above mentioned compounds were performed by MacroModel 10.2 [44,45] at the empirical molecular mechanics (MM) level, with Monte Carlo Multiple Minimum (MCMM) method and Low Mode Conformational Search (LMCS) method. Also, molecular dynamic simulations were performed at 450, 600, 700, 750 K, with a time step of 2.0 fs, an equilibration time of 0.1 ns, and a simulation time of 10 ns. All the produced conformers were then analyzed, and non-redundant conformers were selected by using the “Redundant Conformer Elimination” module of Macromodel 10.2 [44]. Then, the obtained conformers were optimized at quantum mechanical (QM) level by using the MPW1PW91 functional and the 6-31G(d) basis set. The selected conformers accounted for the subsequent computation of the ^13^C and ^1^H NMR chemical shifts, using the MPW1PW91 functional and the 6-31G(d,p) basis set. The final ^13^C NMR and ^1^H NMR spectra were built considering the influence of each conformer on the total Boltzmann distribution and taking into account the relative energies. All the ^13^C and ^1^H NMR calculated chemical shifts were scaled to tetramethylsilane (TMS).

Experimental and calculated ^13^C and ^1^H NMR chemical shifts were compared in detail, computing the Δδ parameter (see Tables S1–S4, Supplementary Materials):

Δδ = |δ_exp_ − δ_calc_|

where, δ_exp_ (ppm) and δ_calc_ (ppm) are the ^13^C/^1^H experimental and calculated chemical shifts, respectively.

The mean absolute errors (MAEs) for all the possible diastereoisomers (see Table 2, Table 3 and Tables S1–S4, Supplementary Materials) were computed:

MAE = ∑(Δδ)/n

defined as the summation through n of the absolute error values (difference of the absolute values between corresponding experimental and ^13^C-^1^H chemical shifts), normalized to the number of the chemical shifts considered.

Furthermore, DP4+ probabilities related to all the considered stereoisomers of **1a**, **1b**, **2-8*R***, **2-8*S*** were computed considering both ^13^C and ^1^H chemical shifts, and they were compared with the related experimental data, using the available DP4+ Toolbox (Excel file). 

Once obtained, the relative configurations of the investigated compounds and the prediction of CD spectra were performed using all the conformers previously obtained from DFT calculations, by submitting them to another round of geometry and energy optimization at DFT, in acetonitrile IEFPCM. Afterwards, QM calculations were performed at TDDFT (NStates = 40) MPW1PW91/6-31g(d,p) level, in acetonitrile IEFPCM to reproduce the experimental solvent environment. The final CD spectra for both the enantiomers related to the determined relative stereoisomers were built considering the influence of each conformer on the total Boltzmann distribution while taking into account the relative energies, and were graphically plotted using SpecDis software [47]. In order to simulate the experimental CD curve, a Gaussian band-shape function was applied with the exponential half-width (σ/γ) of 0.20 eV. All QM calculations were performed using Gaussian 09 software package [48].

### 3.5. Cytotoxicity Assay

HCT116, MCF-7 and SMMC-7721 cell lines were grown in DMEM medium supplemented with 10% FBS (fetal bovine serum), K562 cell line was cultured in RPMI 1640 and supplemented with 10% FBS. All cell lines were incubated at 37 °C with 5% CO_2_ in air atmosphere. The density of tumor cells plated in a 96-well plate was 1 × 10^4^ cells/well. The cells were treated with the isolated compounds in six concentration gradients (threefold dilution starting from 100 μM dissolved in 1% DMSO in the final cell medium), and the cells treated with medium containing 1% DMSO served as a control. Adriamycin was used as positive control. Triplicate wells with untreated cells were set up to serve as a vehicle control. After 48 h, the relative cell viability was assayed by CellTiter-Blue^®^ Cell viability assay kit, and the fluorescence was recorded in a microplate reader (Synergy 2, BioTek Instruments, Inc., Winooski, VT, USA) at 530/590 nm. The IC_50_ was determined by fitting the relative cell viability curve by a dose-response model in the Prism program from GraphPad Software [49]. The in vitro cytotoxicity assay was performed three times with triplicates in each experiment. *p* values were calculated using the Student’s *t* test, and a probability of 0.05 or less was considered statistically significant. IC_50_ results are given as the mean of three independent experiments in triplicate in each experiment.

## 4. Conclusions

A new polyketide, simplextone E (**1**), together with eight known analogues (**2**–**9**), were isolated from the South China Sea sponge *Plakortis* sp. Polyketides of this family contain flexible moieties that may rotate freely around a single bond. Determination of the absolute configuration of these segments is one of the most challenging parts in structural elucidation of natural products. Recently, quantum mechanical calculation has provided novel and reliable approaches to resolve this tricky issue. Calculation for ^13^C and ^1^H NMR chemical shifts gives relative configuration and the TDDFT/ECD calculation leads to the assignment of the absolute configuration. The research gives an example for structure determination of flexible molecules by combination of spectroscopic analysis and quantum mechanical calculation. In the biotest in vitro, compounds **6** and **7** with peroxide moiety showed a potent growth inhibitory effect against HCT-116 cells with similar IC_50_ values as that of the positive control adriamycin, while polyketides with lactone fragment showed weak cytotoxic activity. Compounds **6** and **7** also showed selective activity towards MCF-7 and K562 cells, while compound **3** displayed weak activity against K562 cells. All the tested compounds were inactive towards SMMC-7721 cell lines. The tumor cell growth inhibitory activity of compounds **6** and **7** may be attributed to the peroxide moiety in the structure, as reported previously [50].

## Figures and Tables

**Table 1:** ^1^H (400 MHz) and ^13^C NMR (100 MHz) data for compounds **1** (in CDCl_3_, *J* in Hz).

Position	δ_C_	δ_H_	COSY	HMBC (H→C)
1	175.0, C			
2α 2β	38.1, CH_2_	2.98 dd (18.4, 9.3) 2.56 dd (18.4, 7.0)		1, 4 1
3	72.5, CH	4.90 m	2α, 2β	
4	94.4, C			
5	55.5, CH	2.25 d (7.8)	9	3, 4, 6, 7, 8, 13
6	84.3, C			
7α 7β	48.9, CH_2_	1.43 m 1.93 m		
8	36.4, CH	2.00 m	7β	
9	50.2, CH	1.58 m	10a	
10a 10b	33.5, CH_2_	1.69 m 1.46 m	11	8, 9 8, 9
11	22.3, CH_2_	1.38 m	12	9
12	14.3, CH_3_	0.92 ov		10a, 10b
13a 13b	26.6, CH_2_	2.08 m 1.87 m		4 3, 4
14	8.3, CH_3_	1.05 t (7.3)	13	4
15a 15b	36.4, CH_2_	1.71 m 1.56 m		6, 7 6, 7
16	8.5, CH_3_	0.96 ov	15	6
17	22.0, CH_3_	0.99 d (6.5)	8	7, 8, 9

**Table 2:** The calculation results of compound **1**, with mean absolute errors (MAE) values and DP4+ probabilities.

MAE Values (ppm)				DP4+ Probability		
Isomer	Number of Conformers	^13^C MAE	^1^H MAE	^13^C Data	^1^H Data	All Data
**1a**	15	1.83	0.18	100.00%	2.70%	99.98%
**1b**	16	2.14	0.15	0.00%	97.30%	0.01%

**Table 3:** The calculation results of compound **2**, with MAE values and DP4+ probabilities.

MAE Values (ppm)				DP4+ Probability		
Isomer	Number of Conformers	^13^C MAE	^1^H MAE	^13^C Data	^1^H Data	All Data
**2-8*R***	170	1.52	0.14	61.67%	6.25%	9.69%
**2-8*S***	150	1.44	0.13	38.33%	93.75%	90.31%

**Table 4:** Tumor cell growth inhibitory activity of compounds **1**–**9** (IC_50_ in μM).

	1	2	3	4	5	6	7	8	9	Adriamycin ^a^
HCT-116	>50.0	>50.0	>50.0	>50.0	>50.0	8.3 ± 2.4	8.4 ± 2.3	>50.0	>50.0	4.1
MCF-7	>50.0	>50.0	49.3 ± 3.5	>50.0	>50.0	13.2 ± 1.6	>50.0	>50.0	>50.0	6.2
K562	>50.0	>50.0	>50.0	-	>50.0	25.4 ± 5.4	30.3 ± 3.5	>50.0	-	5.1
SMMC-7721	>50.0	>50.0	>50.0	>50.0	>50.0	>50.0	>50.0	>50.0	>50.0	5.3

^a^ Positive control.

## Supplementary Materials

Spectra of the compound **1** and calculated NMR chemical shifts for compounds **1** and **2** are available online at www.mdpi.com/1660-3397/15/5/129/s1.
