# Supplementary material for: Chemistry and Selective Tumor Cell Growth Inhibitory Activity of Polyketides from the South China Sea Sponge Plakortis sp
Source: Mar Drugs. 2017 May 3;15(5):129. doi: 10.3390/md15050129 (PMC5450535; doi:10.3390/md15050129)
Supplement: Supplementary file 1 [file marinedrugs-15-00129-s001.pdf]

Supplementary Materials

Chemistry and selective tumor cell growth inhibitory activity of polyketides from the South China Sea Sponge *Plakortis* sp.

Jiao Li, [†,1] Cui Li, [†,⊥,1] Raffaele Riccio,[2] Gianluigi Lauro,[2] Giuseppe Bifulco,[2] Tie-Jun Li,[1] Hua Tang,[1] Chun-Lin Zhuang,[1] Hao Ma, [1] Peng Sun,[1] Wen Zhang[*,1]

[1] Research Center for Marine Drugs, School of Pharmacy, Second Military Medical University, 325 Guo-He Road, Shanghai 200433, P. R. China

[2] Dipartimento di Farmacia, Universita' di Salerno, Via Giovanni Paolo II 132 84084 Fisciano (SA), Italy

Index

Figure S1. HRESIMS spectrum of **1**	S2
Figure S2. FTIR of **1**	S2
Figure S3. ^{1}H NMR spectrum of **1** in $CDCl_3$ at 400 MHz	S3
Figure S4. ^{13}C NMR and DEPT spectrum of **1** in $CDCl_3$ at 100 MHz	S3
Figure S5. HSQC spectrum of **1** in $CDCl_3$	S4
Figure S6. ^{1}H-^{1}H COSY spectrum of **1** in $CDCl_3$	S4
Figure S7. HMBC spectrum of **1** in $CDCl_3$	S5
Figure S8. NOESY spectrum of **1** in $CDCl_3$	S5
Figure S9. NOE difference spectrum of **1** in $CDCl_3$	S6
Table S1 ^{13}C experimental and calculated NMR chemical shifts for **1a-b**	S7
Table S2. ^{1}H experimental and calculated NMR chemical shifts for **1a-b**	S8
Table S3. ^{13}C experimental and calculated NMR chemical shifts for **2-8*R***, **2-8*S***	S9
Table S4. ^{1}H experimental and calculated NMR chemical shifts for **2-8*R***, **2-8*S***	S10
Figure S10. Histograms of relative cell viability percentage for compounds **3** and **6** against MCF-7 cell lines.	S11
Figure S11. Histograms of relative cell viability percentage for compounds **6** and **7** against K562 cell lines.	S11

[†] Both are co-first author.

* Corresponding author. Tel: +86 21 81871257; e-mail: wenzhang1968@163.com

[⊥] Present address: Science and Research Laboratory, LonghuaHosptial, Shanghai University of Traditional Chinese Medicine, 725 South Wanping Road, Shanghai 200032, P. R. China.

Data Filename LJ-40.d
Sample Name LJ-40
User Name
Acquired Time 2014-4-25 4:17:29 PM
Instrument
Agilent Technologies 6224 TOF LC/MS

User Spectra

Fragmentor Voltage	Collision Energy	Ionization Mode
150	0	ESI

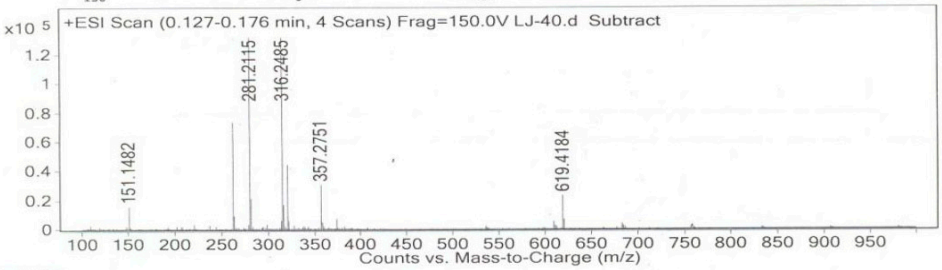

Peak List

m/z	z	Abund	Formula	Ion
151.1482		15702.2		
263.2009	1	74206.9		
281.2115	1	168667.8		
281.247		10404.4		
282.2148	1	21659.7		
316.2485	1	132144.2	C17 H34 N O4	(M+NH4)+
317.2519	1	17142.5	C17 H34 N O4	(M+NH4)+
321.2041		44024.2		
357.2751		30920.1		
619.4184		23204.6		

Formula Calculator Results

IonFormula	Measured Mass	Tgt Mass	Diff (ppm)	Score
C17 H34 N O4	316.2485	316.2482	-1.01	90.86

--- End Of Report ---

Figure S1. HRESIMS spectrum of **1**

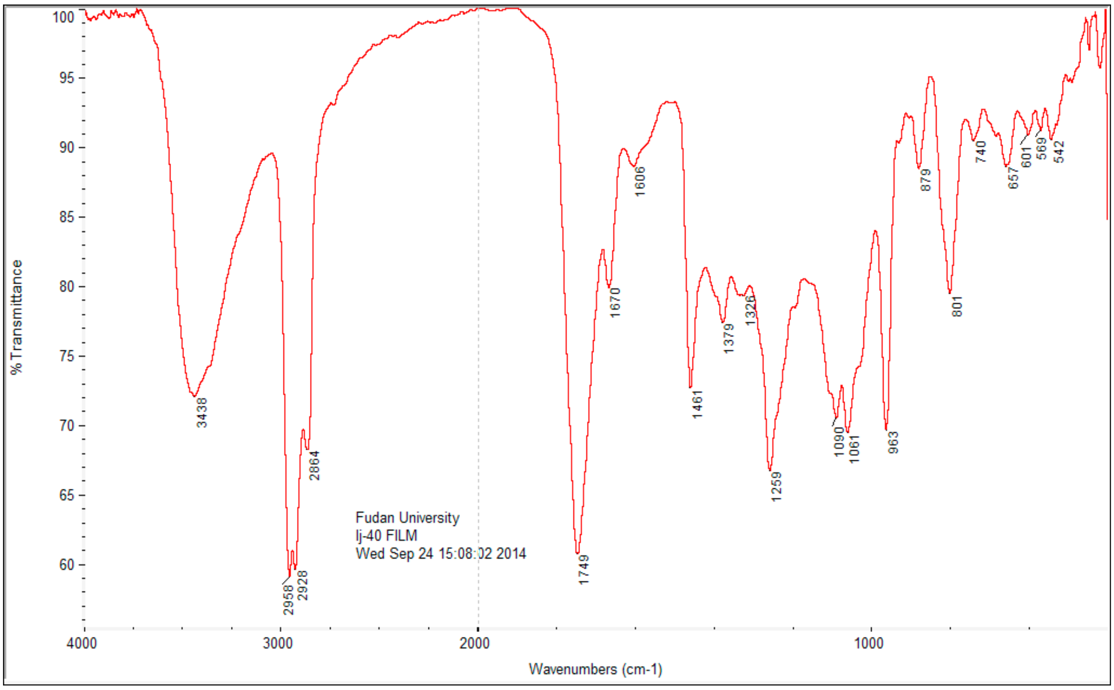

Figure S2. FTIR of **1**

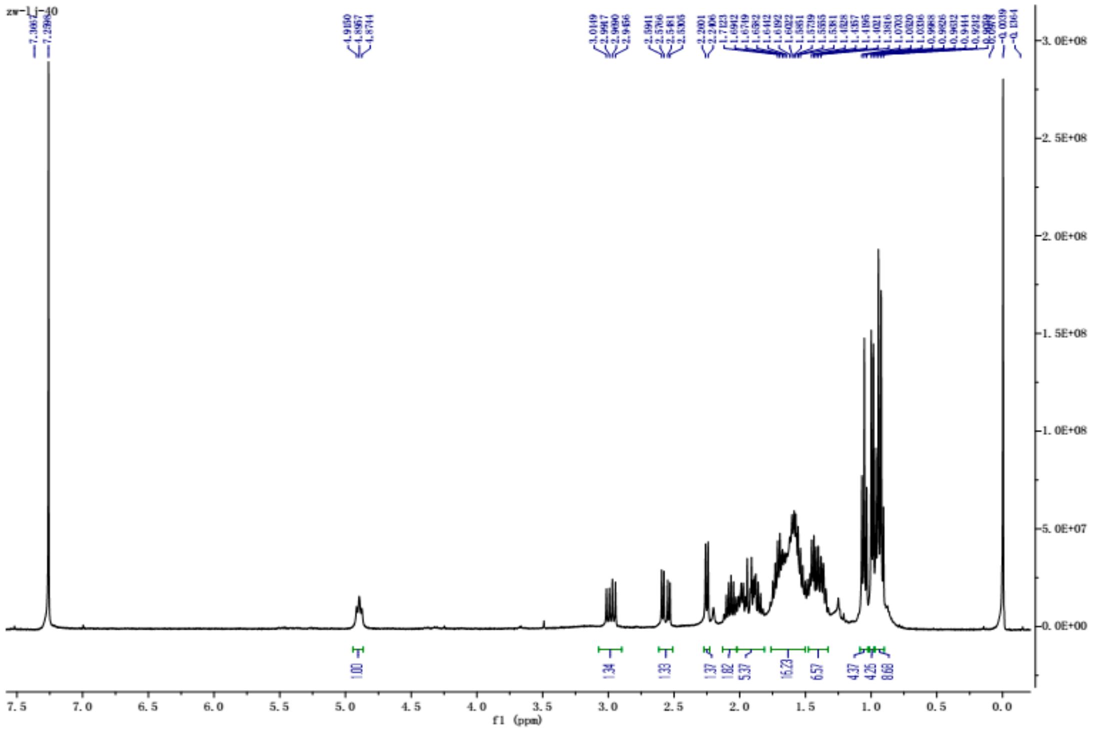

Figure S3. ^{1}H NMR spectrum of **1** in $CDCl_3$ at 400 MHz

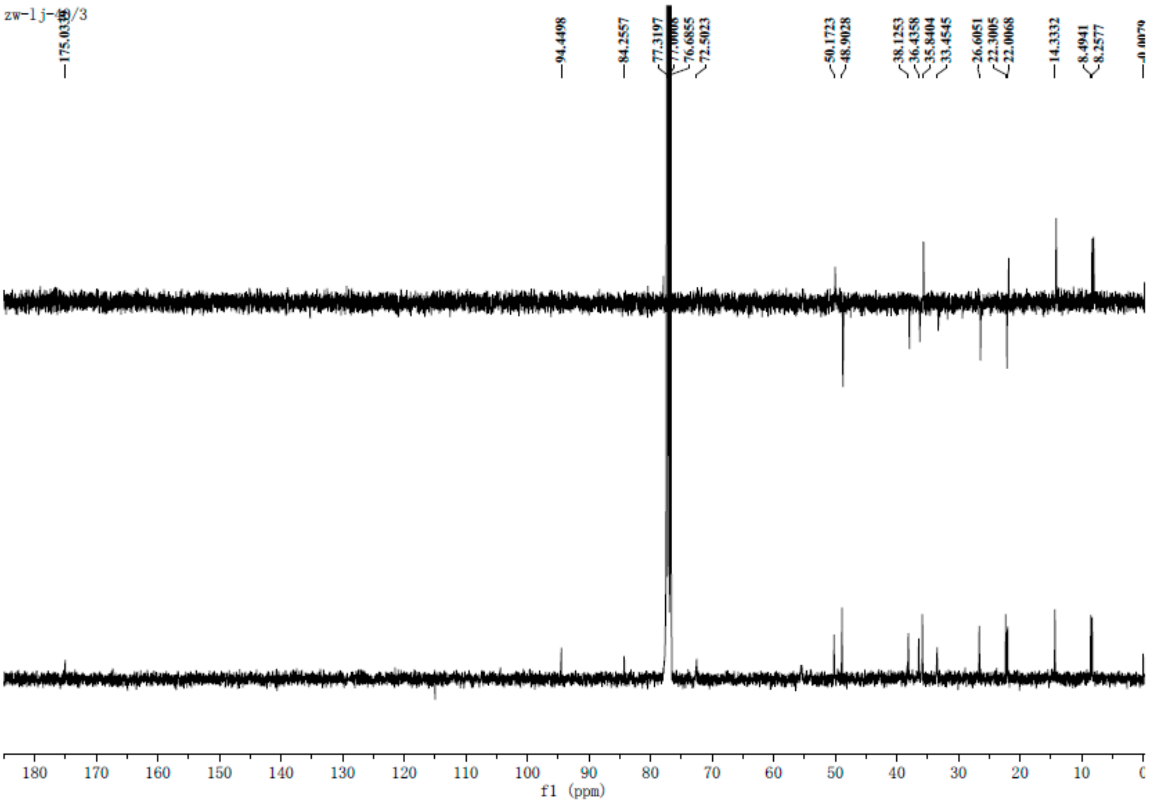

Figure S4. ^{13}C NMR and DEPT spectrum of **1** in $CDCl_3$ at 100 MHz

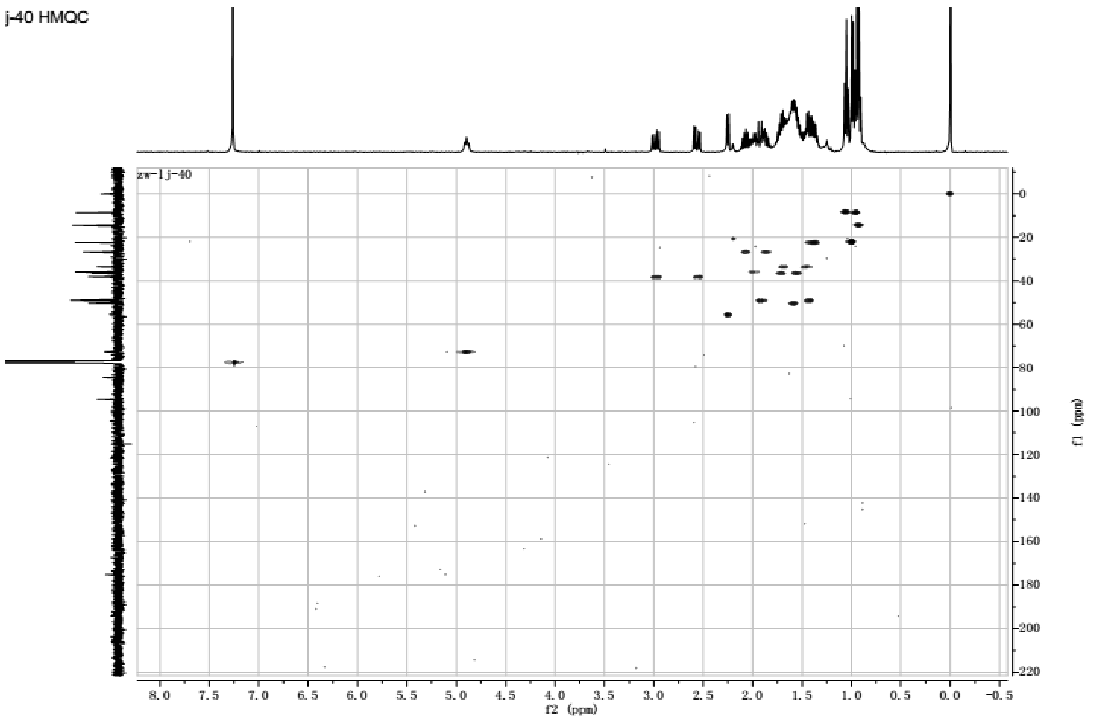

Figure S5. HSQC spectrum of **1** in $CDCl_3$

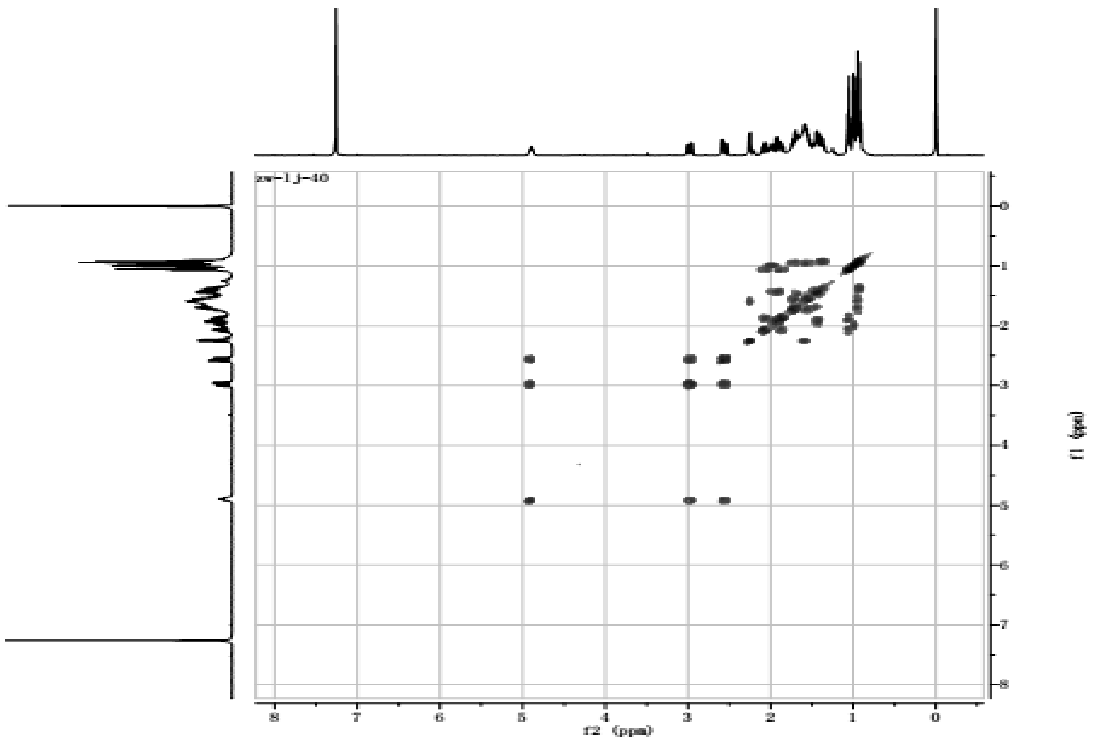

Figure S6. ^1H-^1H COSY spectrum of **1** in $CDCl_3$

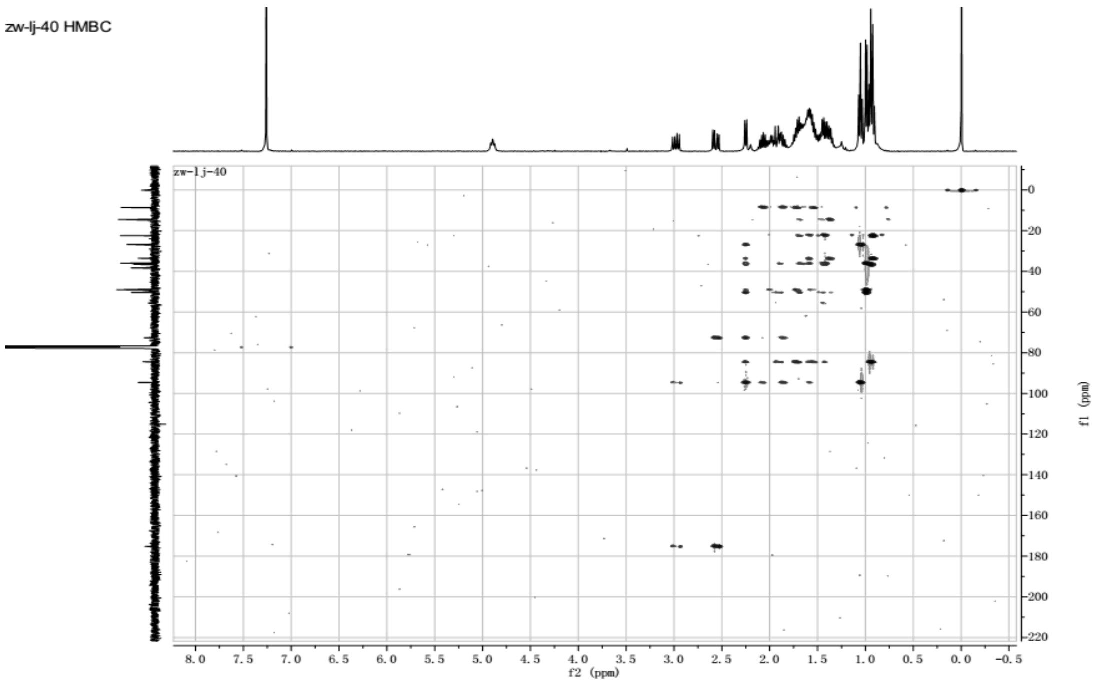

Figure S7. HMBC spectrum of **1** in $CDCl_3$

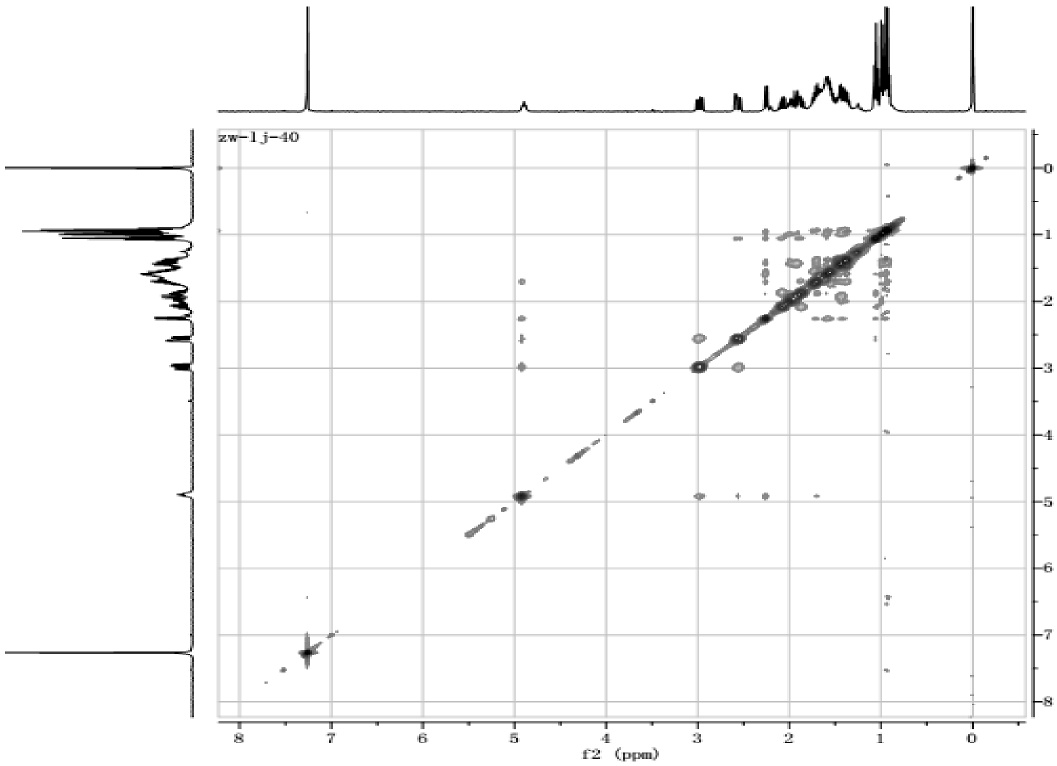

Figure S8. NOESY spectrum of **1** in $CDCl_3$

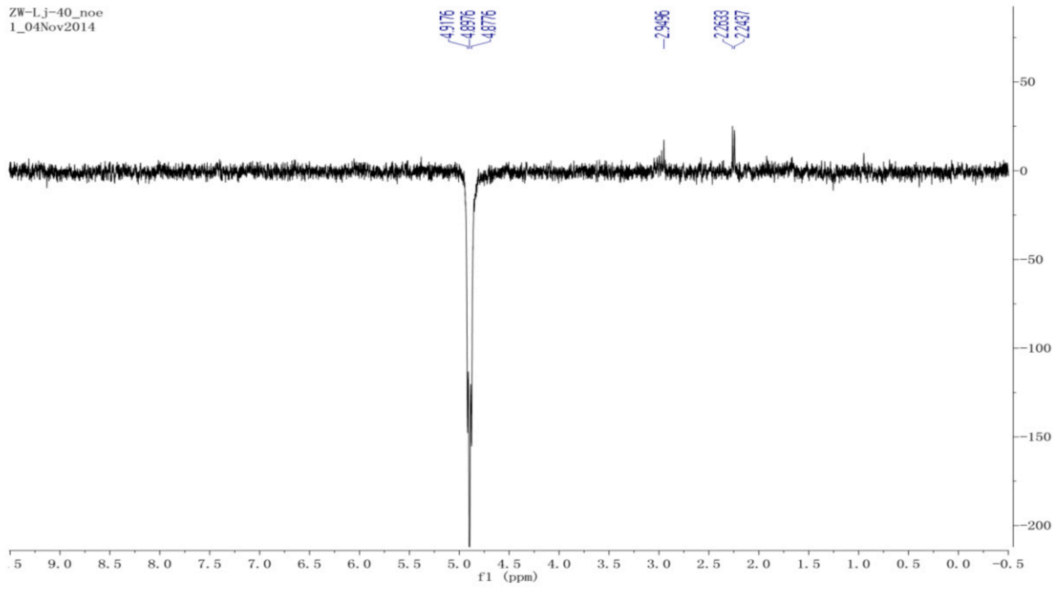

Figure S9. NOE difference spectrum of **1** in $CDCl_3$

Table S1 ^{13}C experimental and calculated NMR chemical shifts for **1a-b**, with [a]|Δδ|(^{13}C) and [b]MAE values. Chemical shift data here reported were produced using tetramethylsilane (TMS) as reference compound.

**δexp (^{13}C), ppm**		**δcalc (^{13}C), ppm**		**|Δδ| (^{13}C), ppm[a]**	
**position**		**1a**	**1b**	**1a**	**1b**
1	175.0	IGNORED	IGNORED	IGNORED	IGNORED
2	38.1	36.9	35.1	1.2	3.0
3	72.5	75.1	69.9	2.6	2.6
4	94.4	89.6	87.5	4.8	6.9
5	55.5	57.6	50.2	2.1	5.3
6	84.3	82.1	82.9	2.2	1.4
7	48.9	49.5	49.4	0.6	0.5
8	36.4	37.2	37.4	0.8	1.0
9	50.2	51.7	50.8	1.5	0.6
10	33.5	32.3	35.9	1.2	2.4
11	22.3	25.3	24.4	3.0	2.1
12	14.3	15.7	16.0	1.4	1.7
13	26.6	28.0	28.7	1.4	2.1
14	8.3	9.7	9.6	1.4	1.3
15	36.4	39.6	35.1	3.2	1.3
16	8.5	10.1	10.5	1.6	2.0
17	22.0	21.8	22.3	0.2	0.3
**MAE[b]**				1.83	2.14

[a] **|Δδ|(^{13}C)** = $|\delta_{exp} - \delta_{calc}|$ (^{13}C), ppm: absolute differences for experimental versus calculated ^{13}C NMR chemical shifts

[b] **MAE** = $\Sigma[|(\delta_{exp} - \delta_{calcd})|]/n$, summation through n of the absolute error values (difference of the absolute values between corresponding experimental and calculated ^{13}C chemical shifts), normalized to the number of the chemical shifts

Table S2. ^{1}H experimental and calculated NMR chemical shifts for **1a-b**, with [a]$|\Delta\delta|(^{1}H)$ and [b]MAE values. Chemical shift data here reported were produced using tetramethylsilane (TMS) as reference compound.

	δexp (^{1}H), ppm	δcalc (^{1}H), ppm		|Δδ| (^{1}H), ppm[a]	
**position**		**1a**	**1b**	**1a**	**1b**
2α	2.98	2.49	2.47	0.49	0.51
2β	2.56	2.42	2.32	0.14	0.24
3	4.90	4.29	4.94	0.61	0.04
5	2.25	2.18	2.58	0.07	0.33
7α	1.43	1.23	1.31	0.20	0.12
7β	1.93	2.15	1.68	0.22	0.25
8	2.00	1.92	2.06	0.08	0.06
9	1.58	1.58	1.76	0.00	0.18
10α	1.69	1.93	1.56	0.24	0.13
10β	1.46	1.30	1.54	0.16	0.08
11	1.38	1.47	1.42	0.09	0.04
12	0.92	0.99	0.92	0.07	0.00
13α	2.08	2.02	2.09	0.06	0.01
13β	1.87	1.99	1.59	0.12	0.28
14	1.05	1.16	1.00	0.11	0.05
15α	1.71	1.92	2.14	0.21	0.43
15β	1.56	1.10	1.48	0.46	0.08
16	0.96	1.02	0.87	0.06	0.09
17	0.99	0.89	0.99	0.10	0.00
**MAE[b]**				0.18	0.15

[a] $|\Delta\delta|(^{1}H) = |\delta_{exp} - \delta_{calc}|$ (^{1}H), ppm: absolute differences for experimental versus calculated ^{1}H NMR chemical shifts; [b] **MAE** = $\Sigma[|(\delta_{exp} - \delta_{calcd})|]/n$, summation through n of the absolute error values (difference of the absolute values between corresponding experimental and calculated ^{1}H chemical shifts), normalized to the number of the chemical shifts

Table S3. ^{13}C experimental and calculated NMR chemical shifts for **2-8*R***, **2-8*S***, with $^{a}|\Delta\delta|(^{13}C)$ and ^{b}MAE values. Chemical shift data here reported were produced using tetramethylsilane (TMS) as reference compound.

	δexp (^{13}C), ppm	δcalc (^{13}C), ppm		|Δδ| (^{13}C), ppm[a]	
**position**		**2-8*R***	**2-8*S***	**2-8*R***	**2-8*S***
1	174.7	IGNORED	IGNORED	IGNORED	IGNORED
2	38.0	38.6	38.4	0.6	0.4
3	77.3	78.5	78.3	1.2	0.9
4	94.9	91.8	91.8	3.1	3.1
5	81.6	82.3	82.7	0.7	1.1
6	88.3	87.0	87.1	1.3	1.2
7	42.4	43.6	43.3	1.2	0.9
8	28.5	30.7	30.3	2.2	1.8
9	38.4	38.7	38.3	0.4	0.0
10	29.2	30.2	30.3	1.0	1.0
11	22.9	25.0	24.9	2.1	2.0
12	14.1	15.9	15.9	1.8	1.8
13	29.2	31.6	31.5	2.4	2.3
14	8.0	9.8	9.9	1.9	1.9
15	26.0	28.7	28.7	2.6	2.7
16	8.2	9.8	10.1	1.6	1.9
17	21.1	21.3	21.2	0.1	0.0
**MAE**[b]				1.52	1.44

[a] $\mathbf{|\Delta\delta|(^{13}C)} = |\delta_{exp} - \delta_{calc}|$ (^{13}C), ppm: absolute differences for experimental versus calculated ^{13}C NMR chemical shifts; [b] **MAE** = $\Sigma[|(\delta_{exp} - \delta_{calcd})|]/n$, summation through n of the absolute error values (difference of the absolute values between corresponding experimental and calculated ^{13}C chemical shifts), normalized to the number of the chemical shifts

Table S4. ^{1}H experimental and calculated NMR chemical shifts for **2-8*R***, **2-8*S***, with [a]|Δδ|(^{1}H) and [b]MAE values. Chemical shift data here reported were produced using tetramethylsilane (TMS) as reference compound.

	**δexp (^{1}H), ppm**	**δcalc (^{1}H), ppm**		**|Δδ| (^{1}H), ppm[a]**	
**position**		**2-8*R***	**2-8*S***	**2-8*R***	**2-8*S***
2α	2.68	2.34	2.35	0.34	0.33
2β	2.78	2.41	2.41	0.37	0.37
3	4.34	4.15	4.15	0.19	0.19
5	3.87	3.94	3.86	0.07	0.01
7α	1.46	1.64	1.43	0.18	0.03
7β	1.36	1.23	1.37	0.13	0.01
8	1.57	1.61	1.73	0.04	0.16
9α	1.34	1.41	1.55	0.07	0.21
9β	1.15	1.15	1.12	0.00	0.03
10	1.26	1.32	1.32	0.06	0.06
11	1.28	1.32	1.33	0.04	0.05
12	0.89	0.93	0.93	0.04	0.04
13α	1.80	1.53	1.53	0.27	0.27
13β	1.93	1.59	1.60	0.34	0.33
14	1.04	1.18	1.15	0.14	0.11
15	1.62	1.51	1.56	0.11	0.06
16	0.92	0.94	0.96	0.02	0.04
17	0.94	1.04	0.98	0.10	0.04
5-OH	2.28	IGNORED	IGNORED	IGNORED	IGNORED
**MAE[b]**				0.14	0.13

[a] **|Δδ|(^{1}H)** = $|\delta_{exp} - \delta_{calc}|$ (^{1}H), ppm: absolute differences for experimental versus calculated ^{1}H NMR chemical shifts; [b] **MAE** = $\Sigma[|(\delta_{exp} - \delta_{calcd})|]/n$, summation through n of the absolute error values (difference of the absolute values between corresponding experimental and calculated ^{1}H chemical shifts), normalized to the number of the chemical shifts.

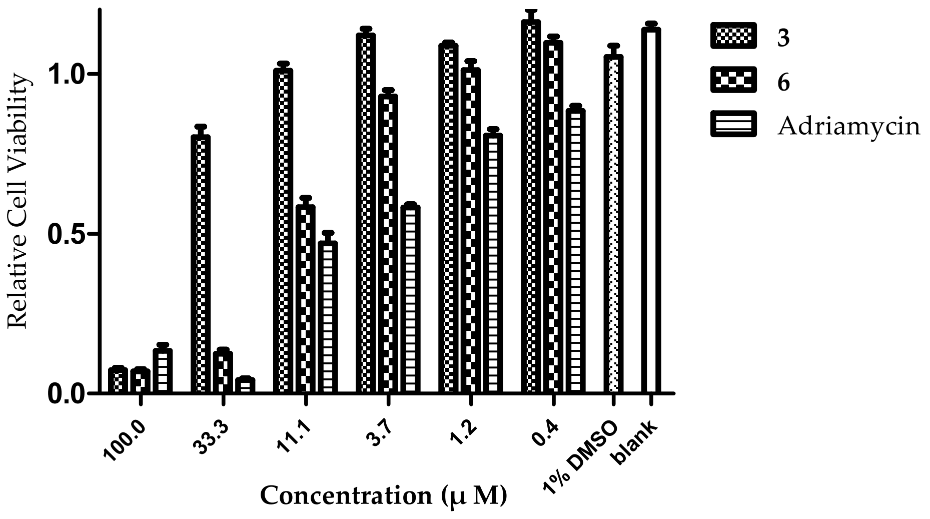

Figure S10. Histograms of relative cell viability percentage for compounds **3** and **6** against MCF-7 cell lines.

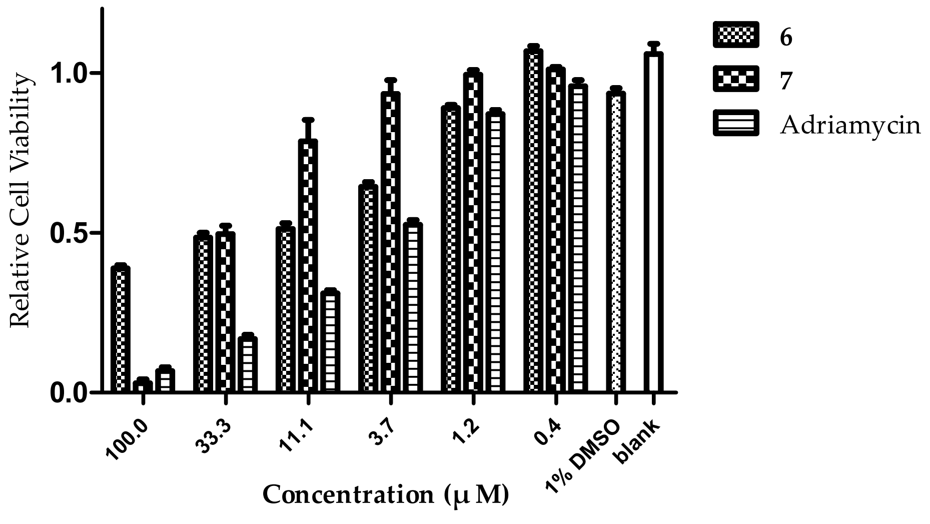

Figure S11. Histograms of relative cell viability percentage for compounds **6** and **7** against K562 cell lines.
